# Professional Grief Among Psycho‐Oncologists in Germany: A Cross‐Sectional Survey Study

**DOI:** 10.1002/pon.70355

**Published:** 2025-12-05

**Authors:** Svenja Wandke, Klaus Lang, Martin Härter, Karin Oechsle, Carsten Bokemeyer, Mareike Rutenkröger, Isabelle Scholl

**Affiliations:** ^1^ Department of Medical Psychology Center for Psychosocial Medicine University Medical Center Hamburg‐Eppendorf Hamburg Germany; ^2^ II. Department of Medicine University Medical Center Hamburg‐Eppendorf Hamburg Germany; ^3^ Psychotherapeutic Practice Munich Germany

**Keywords:** cancer, oncology, patient death, professional grief, psycho‐oncology

## Abstract

**Background:**

Professional grief refers to the emotional response healthcare professionals may experience following patient deaths. Although likely relevant in clinical practice, this phenomenon has been overlooked in research—particularly among psycho‐oncologists. This study examined the emotional impact, coping strategies, and support needs related to professional grief in a German sample of psycho‐oncologists.

**Methods:**

A cross‐sectional online survey was disseminated via professional associations and randomly selected cancer centers in Germany. Eligible participants were working in psycho‐oncology and had experienced at least one patient death. The survey included two established instruments (Texas Revised Inventory of Grief—Present Feelings [TRIG‐D], Professional Bereavement Scale [PBS‐D]) and self‐developed items on emotional responses, coping behaviors, and support needs.

**Results:**

258 participants (91% female; mean age 48 years) were included. Participants reported an average of three patient deaths per month and moderate overall distress (scale 0–10, *M* = 5.02, SD = 2.14). Scores on both grief scales were in the low to moderate range. Most participants reported positive effects of professional grief on for example their sense of purpose, with minimal impact on relationships. Common coping strategies included adopting an accepting stance and peer support. Unmet support needs were identified, particularly regarding professional training and timely communication about patient deaths.

**Conclusion:**

This study offers the first quantitative insight into professional grief among psycho‐oncologists in Germany. Professional grief levels were low to moderate. The reported positive changes suggest that patient deaths may even pose a chance for professional growth. Future research should explore relevant risk and protective factors to guide targeted support.

## 1 Background

Grief is commonly understood as a response to the loss of someone with whom a meaningful bond existed [1]. Traditional perspectives on grief predominantly focus on personal rather than professional relationships between healthcare professionals (HCPs) and patients [2]. In cancer care, patient loss is frequent and emotionally charged for HCPs [3]. Nevertheless, HCPs often develop significant connections with patients [4]. Recent evidence suggests that grief experienced in the professional context—termed professional grief—differs from personal grief in important ways, though the boundary is not always clear [2]. In a recent scoping review, we outlined core features of professional grief in oncology, including its frequent occurence, lower intensity, and confinement to the professional setting [6]. It may involve emotions such as guilt or a sense of responsibility and is often perceived as inappropriate within professional or societal norms [5].

Patient deaths can considerably impact HCPs' mental health and well‐being. Previous research links professional grief to adverse outcomes including declining mental health [6], reduced well‐being and quality of life [2, 4, 6, 7], and compromised patient care [8, 9]. While most research has focused on nurses and physicians [5], psycho‐oncologists are also closely involved in multidisciplinary cancer care, in line with national and international guidelines [10, 11, 12]. Psycho‐oncologists, by definition, provide psychosocial support to individuals facing serious illness and end‐of‐life situations [13]. Their work is grounded in sustained therapeutic relationships [14]. These emotionally intense interactions—frequently centered around existential concerns and psychological well‐being—can foster strong interpersonal bonds that may increase vulnerability to professional grief [2, 15]. Despite the clinical importance and emotional complexity of their work, there is a lack of empirical data on professional grief among psycho‐oncologists, particularly within European healthcare contexts. The existing literature is heavily skewed toward Anglo‐American settings, and studies from Germany are absent [5].

Few studies have used quantitative methods to examine professional grief, limiting generalizability [5]. To date, only one quantitative study has investigated professional grief among psycho‐oncologists, based on an Israeli sample [2], reporting moderate grief levels and a link between high grief (especially with low social acknowledgment) and compassion fatigue. In Germany, only one qualitative interview study [16] has explored this topic, revealing both negative (e.g., loss of spiritual beliefs) and positive effects of patient deaths, such as heightened alignment with personal values and development of a “routine” to cope with death, elevating composure. It also identified peer support as central and unmet needs regarding emotional support and training. No study has yet addressed all three evidence gaps: (1) European samples, (2) psycho‐oncologists as a distinct group, and (3) quantitative data. This cross‐sectional study addresses these gaps by examining professional grief among German psycho‐oncologists. We aimed to: (1) characterize the experience of professional grief, including emotional responses, influencing factors, and impacts on personal and professional life; (2) identify coping strategies and support resources; and (3) assess unmet support needs.

## 2 Methods

### 2.1 Study Design

This study employed a cross‐sectional design using an anonymous, one‐time online survey. The study was conducted and reported in accordance with the STROBE (Strengthening the Reporting of Observational Studies in Epidemiology) guidelines [17] (Supporting Information S1). Due to its specificity to online survey studies, the CHERRIES (Checklist for Reporting Results of Internet E‐Surveys) checklist [18] was used additionally (Supporting Information S2). Generative AI (e.g., ChatGPT) assisted with manuscript structure, formating and language editing. All content was author‐verified.

#### 2.1.1 Sampling and Recruitment

Participants were recruited via several professional organizations (Supporting Information S3). These organizations distributed the survey invitation containing a link to participate via email and/or newsletter between August and September 2024. The survey remained open for participation from August until November 2024. To supplement this recruitment strategy, contact details of psycho‐oncological services from cancer centers were identified using the online directory of cancer care facilities certified by the German Cancer Society (“Oncomap”). From this database, 180 facilities were systematically selected by choosing every fifth center listed within each federal state for the three cancer entities with the highest absolute numbers of deaths in Germany (lung, pancreatic, and breast cancer) [19].

As an incentive, participants were given the opportunity to enter a raffle for one of 20 online vouchers, each valued at €15.

The estimated total population of psycho‐oncologists in Germany was derived from Schulz et al. [20] and set at *N* = 3183. Based on this figure, the minimum required sample size was calculated to be 93, assuming a margin of error (*e*) of 0.1, a proportion (*p*) of 0.5, and a z‐score of 1.96. The formula used for this calculation was: $\frac{\frac{z^{2}\times p\left(1-p\right)}{e^{2}}}{1+\left(\frac{z^{2}\times p\left(1-p\right)}{e^{2}N}\right)}$ [21]. Inclusion criteria specified that participants had to provide psychosocial care to cancer patients and have experienced the death of at least one patient. Formal training as a psycho‐oncologist by completing a course accredited by the German Cancer Association was not required.

### 2.2 Data Collection

An open online survey was administered via LimeSurvey [22]. Before participation, all respondents provided electronic informed consent after being informed of the study's voluntary and anonymous nature. The complete questionnaire is available in Supporting Information S4.

### 2.3 Measures and Instruments

Our assessment strategy comprised three complementary approaches to capture professional grief comprehensively: (1) a global distress rating on overall burden, (2) two established instruments measuring specific dimensions of grief and bereavement, and (3) context‐specific items developed for this study to assess experiences, coping, and needs particular to psycho‐oncologists. We conceptualize overall distress as participants' subjective global emotional burden from patient deaths, measured through a single item. Grief‐related distress, assessed via the German adaptation of the Texas Revised Inventory of Grief—Present Feelings Subscale (TRIG‐D) [23], captures specific grief symptoms following loss in general. Professional bereavement reactions, measured by the German version of the Professional Bereavement Scale (PBS‐D) [24], include immediate responses to recent patient deaths and accumulated changes over time resulting from repeated exposure to patient loss.

#### 2.3.1 Validated Instruments

##### 2.3.1.1 Texas Revised Inventory of Grief—Present Feelings (TRIG‐D)

Grief‐related distress was measured via the German adaptation of the Texas Revised Inventory of Grief—Present Feelings Subscale [23], comprising 16 items (A3, Supporting Information S4). Responses were recorded on a 5‐point Likert scale ranging from 1 = “completely false” to 5 = “completely true”, with higher scores indicating greater emotional burden. The TRIG‐D has demonstrated sound psychometric properties, with good internal consistency Cronbach's *α* = 0.87 and convergent construct validity [23], although no validated cut‐off scores exist.

##### 2.3.1.2 Professional Bereavement Scale (PBS‐D)

Additionally, we included the Professional Bereavement Scale (PBS) [24], comprising of two subscales: Short‐Term Bereavement Reactions (SBR; items 1–17) and Accumulated Global Changes (AGC; items 18–32), both in Section A4 of Supporting Information S4. Participants rated SBR items by recalling their most recent patient loss and assessing emotional reactions within the first week post‐loss on a 5‐point Likert scale from 0 = “not at all” to 4 = “extremely strong”. For the AGC subscale, they indicated the extent of perceived long‐term personal change using a 5‐point scale from 0 = “No (no such change or the change was not induced by experiencing patient deaths)” to 4 = “Yes, a great deal”. The original English version demonstrated strong psychometric properties (SBR *α* = 0.96, AGC *α* = 0.94) with good construct validity [24]. Since no validated German version existed, we translated and culturally adapted the PBS using the TRAPD methodology [25]. The German version (PBS‐D) used in this study demonstrates acceptable internal consistency (Cronbach's *α* > 0.80 for both subscales). Currently no further psychometric evaluation of the PBS‐D has been concluded so far.

#### 2.3.2 Self‐Developed Items

Given the lack of validated instruments assessing the specific experiences, coping strategies, and support needs of psycho‐oncologists in the German context, we developed additional items based on three complementary sources: (1) themes emerging from our qualitative interview study with German psycho‐oncologists [16], (2) identified domains in our scoping review of professional grief in cancer care [5], and (3) established assessment approaches from international literature [2, 6].

The development process involved multiple stages. First, items were drafted by the research team based on the empirical and theoretical foundations described above. Second, all items underwent review by the full author team, which included experienced psycho‐oncologists (IS, KL, SW, MR, MH) and a palliative care specialist (KO), ensuring clinical relevance and face validity. Additionally, the complete questionnaire was pilot‐tested by an independent psycho‐oncologist for comprehensibility.

##### 2.3.2.1 Section A: Experience of Professional Grief

Section A of the survey focused on the experience of professional grief. In A1, participants were asked to rate the general level of distress they experienced in response to patient deaths, ranging from 0 = “not distressed at all” to 10 = “very distressed.” A2 assessed participants' agreement with a series of statements concerning their emotional and professional attitudes toward patient death. Items were rated on a 5‐point Likert scale from 1 = “strongly disagree” to 5 = “strongly agree”.

In A5 participants reflected on their experienced most stressful patient death and rated the intensity of emotional responses (0 = “not at all” to 4 = “very intense”). A6 consisted of checklist‐style items on contextual distress factors. A7, adapted from Delafontaine et al. [6], assessed perceived impact of recent patient deaths across life domains (−2 = “strong negative impact” to 2 = “strong positive impact”). In A8, participants indicated how long such effects typically lasted (“a few moments to an hour” to “more than a year”).

##### 2.3.2.2 Section B: Coping With Professional Grief

Section B addressed how psycho‐oncologists cope with patient deaths. In B1, participants rated the perceived importance of various coping strategies on a 5‐point Likert scale from 1 = “not important at all” to 5 = “very important.” B2 focused on the personal relevance of different support figures in the coping process, using the same rating scale.

##### 2.3.2.3 Section C: Unmet Needs

Section C focused on unmet needs related to coping with patient deaths. In C1, participants indicated their level of agreement with a series of statements about such unmet needs, using a 5‐point Likert scale. C2 provided an opportunity for participants to elaborate on additional needs or provide further comments in open‐ended text fields.

##### 2.3.2.4 Section D: Demographic Information

Section D gathered demographic information.

## 3 Statistical Analysis

All data were analyzed using IBM SPSS Statistics Version 29 [26]. Item‐level analyses included all participants who fulfilled the inclusion criteria. Descriptive statistics, including frequencies and percentages, were calculated at item level. For scale‐based analyses, only cases in which participants completed at least 70% of the items within a given scale were included, in accordance with best practice recommendations and the scales original authors [24, 27, 28]. A detailed overview of excluded and imputed cases per scale is provided in Supporting Information S5. For each included scale, descriptive statistics such as mean and standard deviation were computed.

To explore potential risk or protective factors, we performed exploratory post‐hoc subgroup analyses of overall distress and TRIG‐D‐measured grief. Subgroups were defined based on (1) the number of colleagues participants work with, due to the known relevance of peer support [5, 16, 29], and (2) their years of professional experience, since prior research identified professional inexperience as a potential risk factor [29, 30, 31]. Given the uneven subgroup sizes and non‐normal data distribution, we used Kruskal‐Wallis tests to examine whether differences between subgroups reached statistical significance (*α* = 0.05).

## 4 Results

### 4.1 Sample Characteristics

**FIGURE 1:**
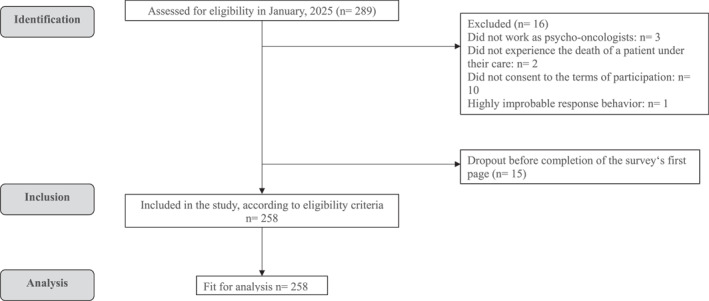
Flow of participants.

A total of *N* = 289 individuals accessed the survey link. Of these, 30 participants were excluded due to reasons such as lack of informed consent or failure to meet inclusion criteria (for a detailed overview, see Figure 1, flow of participants). Additionally, we excluded one case due to implausible and incomplete response patterns. Thus, *N* = 258 participants were deemed eligible for analysis.

A detailed overview of sample characteristics is presented in Table 1. Participants had a mean age 48 years (SD = 12.27), were predominantly female (91.1%) and reported caring for *M* ≈ 36 patients per month (range: 0–200), while experiencing *M* ≈ 3 patient deaths per month (range: 0–25).

**TABLE 1:** Sample characteristics.

Characteristic	
Gender^a^, *n* (%)	
Female	204 (91.1%)
Male	19 (8.5%)
Non‐binary	1 (0.4%)
Age in years, mean (SD)	48.33 (12.27)
Psycho‐oncological care, mean (SD)	
Patients per month	35.6 (25.79)
Patient deaths per month	2.9 (3.65)
Professional background^a^, *n* (%)	
Psychology	154 (68.8%)
Medicine	18 (8.0%)
Social work	12 (5.4%)
Social education/pedagogy	18 (8.0%)
Other	22 (9.8%)
Work setting^b^, *n* (%)	
Outpatient psycho‐oncological services at a hospital	49 (19.0%)
Inpatient psycho‐oncological consultation service at a hospital	100 (38.8%)
Inpatient psycho‐oncological liaison service of at a hospital	64 (24.8%)
Cancer counseling center	53 (20.5%)
Outpatient psychotherapeutic practice	40 (15.5%)
Partial inpatient or inpatient psycho‐oncological services at a hospital	54 (20.9%)
Partial inpatient or inpatient palliative care	51 (19.8%)
Outpatient palliative care	9 (3.5%)
Psycho‐oncological services in a rehabilitation clinic	2 (0.8%)
Professional experience (in years)^a^, *n* (%)	
< 1	5 (2.2%)
1– < 5	59 (26.3%)
5–10	51 (22.8%)
11–15	44 (19.6%)
16–20	24 (10.7%)
> 20	41 (18.3%)
Number of colleagues^c^, *n* (%)	
None	19 (8.5%)
1–5	138 (61.9%)
6–10	30 (13.5%)
> 10	36 (16.1%)
Received formal training in psycho‐oncology^a^, *n* (%)	
Yes	186 (83.0%)
No	38 (17.0%)

^a^ *n* = 224.

^b^ Multiple selection was possible, *n* = 258.

^c^ *n* = 223.

### 4.2 The Experience of Professional Grief

Overall, participants reported a moderate level of distress related to patient deaths, with an average rating of *M* = 5.02 (SD = 2.14) on a scale from 1 (“not distressed at all”) to 10 (“very distressed”). This finding is further supported by the grief‐related measures. Although no validated cut‐off scores are currently available for the PBS‐D or TRIG‐D, the observed values can be cautiously interpreted in relation to the possible score ranges, suggesting overall low to moderate levels of professional grief in this sample. With a mean sum score of *M* = 18.54 (SD = 8.52; range [0–68]), short‐term bereavement reactions measured by the appropriate subscale of the PBS‐D appeared rather limited. Similarly, low levels of grief were observed using the TRIG‐D, with a mean score of *M* = 24.65 (SD = 6.17; range [16–80]). However, the second subscale of the PBS‐D revealed moderate levels of accumulated global changes, with a mean score of *M* = 28.71 (SD = 11.01; range [0–60]).

The intensity of emotional responses varied considerably when participants reflected on their most stressful patient death experience (Table 2). The emotions reported with the highest mean intensity were sorrow/regret (*M* = 2.74, SD = 0.92), grief (*M* = 2.27, SD = 1.01), sadness (*M* = 1.77, SD = 1.05), and helplessness (*M* = 1.57, SD = 1.23). Among these, helplessness showed the greatest variability, with responses distributed almost evenly across all levels of intensity, indicating a wide range of individual experiences (SD = 1.23). In contrast, emotions such as indifference (*M* = 0.15, SD = 0.48), shame (0.21, SD = 0.58) or guilt (0.24, SD = 0.61) were reported as either not experienced or experienced at low intensity by most participants (> 90%).

**TABLE 2:** Emotional impact. Ranking of the emotional response to most stressful patient death, with emotions rated as most intensely felt being at the top.

Emotion	Mean (SD)	Not at all	A little	Some	Intense	Very intense
		(0)	(1)	(2)	(3)	(4)
Frequencies (%)						
Sorrow/regret	2.74 (0.92)	5 (2.1%)	16 (6.7%)	62 (25.9%)	110 (46.0%)	46 (19.2%)
Grief	2.27 (1.01)	15 (6.3%)	31 (13.0%)	89 (37.2%)	83 (34.7%)	21 (8.8%)
Sadness	1.77 (1.05)	29 (12.1%)	64 (26.8%)	90 (37.7%)	44 (18.4%)	12 (5.0%)
Helplessness	1.57 (1.23)	64 (26.8%)	51 (21.3%)	60 (25.1%)	52 (21.8%)	12 (5.0%)
Relief	1.36 (1.01)	60 (25.1%)	65 (27.2%)	84 (35.1%)	28 (11.7%)	2 (0.8%)
Overwhelm	1.18 (1.15)	85 (35.6%)	71 (29.7%)	45 (18.8%)	30 (12.6%)	8 (3.3%)
Apprehension/concern	1.00 (1.02)	101 (42.3%)	65 (27.2%)	46 (19.2%)	25 (10.5%)	2 (0.8%)
Satisfaction	0.88 (1.00)	115 (48.1%)	55 (23.0%)	52 (21.8%)	16 (6.7%)	1 (0.4%)
Fright	0.83 (1.09)	123 (51.5%)	66 (27.6%)	26 (10.9%)	15 (6.3%)	9 (3.8%)
Anger	0.69 (1.00)	139 (58.2%)	59 (24.7%)	21 (8.8%)	16 (6.7%)	4 (1.7%)
Despair	0.39 (0.75)	175 (73.5%)	40 (16.8%)	17 (7.1%)	5 (2.1%)	1 (0.4%)
Guilt	0.24 (0.61)	199 (83.3%)	25 (10.5%)	13 (5.4%)	1 (0.4%)	1 (0.4%)
Shame	0.21 (0.58)	205 (86.1%)	17 (7.1%)	14 (5.9%)	2 (0.8%)	0 (0%)
Indifference	0.15 (0.48)	212 (88.7%)	19 (7.9%)	6 (2.5%)	2 (0.8%)	0 (0%)

*Note: n* = 239.

#### 4.2.1 Factors Influencing the Degree of Perceived Distress

Drawing on prior research [5, 16], this study explored specific patient characteristics, psycho‐oncologist traits, and aspects of the therapeutic relationship that may intensify distress following the death of a patient. Several factors were identified by the majority of participants (> 50%) as contributing to heightened emotional burden. These included situations in which the deceased patient had *underage children* (71.3%), *a long duration of the therapeutic relationship* (67.8%), *young age of the deceased patient* (66.7%), or the *relationship was experienced as especially intense or deep* (64.7%). Additional distress was reported when participants identified themselves strongly with the patient due to *personal similarities*, such as similar age or background (58.5%), or when the *patient's death was perceived as particularly agonizing* (57.8%). In contrast, the timing or manner in which psycho‐oncologists were informed about a patient's death was not seen as a contributor to distress by most participants (Supporting Information S6: Table 1).

### 4.3 Impact Across Different Domains and Duration of Effects Related to Patient Deaths

Most participants reported that their exposure to patient deaths had no effect on their leisure activities (68.9%), patient relationships (62.3%), and personal relationships (57.5%) (Supporting Information S6: Table 2). Additionally, a substantial minority reported a positive or strongly positive impact on personal (36.9%) and patient relationships (36%). Adding to that, a majority of participants indicated that patient deaths had a positive or a strongly positive impact on their sense of purpose (74.1%), their personal representation of life (77.2%) or death (63.5%). Nevertheless, a notable subgroup of 64 participants (28%) reported negative consequences in at least one of all assessed domains. Regarding the duration of these effects, the majority of participants (80.2%) indicated that the impact of patient deaths lasted no longer than 1 week, with 32.2% indicating impacts lasting a few hours up to a day and 40.5% stating to perceive the impact of patient deaths a few days up to a week. Only a very small proportion (1.3%) reported that the consequences persisted for more than 1 year (Supporting Information S6: Table 3).

### 4.4 Coping With Professional Grief

Overall, participants rated most of the proposed coping mechanisms as important (Table 3). On a scale from 1 (not important at all) to 5 (very important), the highest‐rated strategies were: *adopting an accepting attitude toward death* (*M* = 4.57), *informal exchange with colleagues* (*M* = 4.53), *commemorating the deceased patient* (*M* = 4.34), *formal exchange with colleagues* (*M* = 4.24), *expression of one's own emotions* (emotional relief; *M* = 4.23), *finding a positive balance* (*M* = 4.17), *reflection on death and dying* (*M* = 4.07), and *exercise* (*M* = 3.98). *Faith* was the overall lowest rated coping strategy (*M* = 2.79), even though approximately a third of all participants reported that faith is an important or very important coping strategy when faced with patient deaths.

**TABLE 3:** Coping strategies. Ranking of strategies applied by participants to cope with patient deaths, with strategies rated as most important being at the top.

Coping strategy	Mean (SD)	Not important at all	Rather unimportant	Neither important nor unimportant	Rather important	Very important
		(1)	(2)	(3)	(4)	(5)
Frequencies (%)						
Adopting an accepting stance toward the death of people	4.57 (0.62)	1 (0.4%)	1 (0.4%)	6 (2.7%)	78 (34.7%)	139 (61.8%)
Sharing with colleagues (informal, e.g., during the lunch break)	4.53 (0.76)	2 (0.9%)	6 (2.7%)	7 (3.1%)	65 (28.9%)	145 (64.4)
Commemorating the deceased patient	4.34 (0.68)	1 (0.4%)	2 (0.9%)	14 (6.2%)	110 (48.9%)	98 (43.6%)
Sharing with colleagues (formal, e.g., as part of super‐ or intervision)	4.24 (0.90)	1 (0.4%)	15 (6.7%)	19 (8.4%)	85 (37.8%)	105 (46.7%)
Expression of own emotions (emotional relief)	4.23 (0.77)	0 (0%)	8 (3.6%)	22 (9.8%)	106 (47.1%)	89 (39.6%)
Finding a positive balance (e.g., vacations, spending time with friends, reading happy books)	4.17 (1.04)	8 (3.6%)	13 (5.8%)	18 (8.0%)	80 (35.6%)	106 (47.1%)
Reflecting on the topics of death/dying	4.07 (0.91)	3 (1.3%)	13 (5.8%)	29 (12.9%)	100 (44.4%)	80 (35.6%)
Exercise (e.g., sport, yoga or going for a walk)	3.98 (1.09)	7 (3.1%)	23 (10.2%)	23 (10.2%)	86 (38.2%)	86 (38.2%)
Dealing with one's own mortality	3.96 (1.01)	4 (1.8%)	20 (8.9%)	34 (15.1%)	89 (39.6%)	78 (34.7%)
Taking a break from work	3.95 (1.00)	5 (2.2%)	17 (7.6%)	38 (16.9%)	90 (40.0%)	75 (33.3%)
Farewell rituals (e.g., lighting a candle)	3.72 (1.24)	17 (7.6%)	24 (10.7%)	38 (16.9%)	72 (32.0%)	74 (32.9%)
Physical distance from the workplace	3.56 (1.32)	27 (12.0%)	26 (11.6%)	27 (12.0%)	84 (37.3%)	61 (27.1%)
Exchange in a private environment	3.26 (1.06)	11 (4.9%)	52 (23.1%)	49 (21.8%)	94 (41.8%)	19 (8.4%)
Distraction (e.g., reading or watching TV)	3.26 (1.21)	25 (11.1%)	29 (12.9%)	71 (31.6%)	63 (28.0%)	37 (16.4%)
Individual supervision	3.22 (1.22)	22 (9.8%)	43 (19.1%)	60 (26.7%)	63 (28.0%)	37 (16.4%)
Faith	2.79 (1.43)	63 (28.0%)	38 (16.9%)	38 (16.9%)	56 (24.9%)	30 (13.3%)

*Note: n* = 239.

Regarding sources of support, 92% of participants considered colleagues to be at least an important resource in coping with patient deaths, with more than half (55.6%) even rating them as very important. Partners (68%) and friends (47.3%) were also commonly perceived as important sources of support (Supporting Information S6: Table 4).

### 4.5 Psycho‐Oncologists Unmet Support Needs

Participants' responses showed high variability regarding their needs to effectively cope with patient deaths, with answers distributed across the full range of possible response options (Table 4) Regarding number of colleagues, only minor differences in grief and overall distress were obser. However, two notable trends were identified: 69.6% of participants expressed a desire to be *informed about patient deaths as soon as possible*, and 53.6% indicated a preference for being *informed personally or* via *telephone*.

**TABLE 4:** (Unmet) needs. Ranking of needs in regard to coping with patient deaths, with statements most agreed with being at the top.

Need	Mean (SD)	Strongly disagree	Mostly disagree	Neutral	Mostly agree	Strongly agree
		(1)	(2)	(3)	(4)	(5)
Frequencies (%)						
I would like to be informed about the death of patients as soon as possible.	3.76 (1.13)	16 (7.1%)	14 (6.3%)	38 (17.0%)	95 (42.4%)	61 (27.2%)
I would like to be informed personally or by telephone about the death of patients.	3.34 (1.34)	33 (14.7%)	27 (12.1%)	44 (19.6%)	71 (31.7%)	49 (21.9%)
I don't have any unmet needs myself about how I deal with the death of a cancer patient.	3.14 (1.19)	22 (9.8%)	53 (23.7%)	46 (20.5%)	78 (34.8%)	25 (11.2%)
I would like to be better prepared for the death of patients and how to deal with it as part of my professional training.	3.04 (1.18)	25 (11.2%)	51 (22.8%)	63 (28.1%)	61 (27.2%)	24 (10.7%)
I would like to see more rituals of leave‐taking to deceased patients in my workplace.	2.89 (1.22)	39 (17.4%)	44 (19.6%)	63 (28.1%)	59 (26.3%)	19 (8.5%)
I would like to have specific opportunities to talk exclusively about the experience and coping with the death of cancer patients.	2.88 (1.26)	41 (18.3%)	50 (22.3%)	47 (21.0%)	67 (29.9%)	19 (8.5%)
I would like more team supervision to be able to talk about the death of cancer patients.	2.83 (1.24)	38 (17.0%)	61 (27.2%)	47 (21.0%)	58 (25.9%)	20 (8.9%)
I would like more space to talk about how I am affected by the death of cancer patients.	2.75 (1.14)	28 (12.5%)	81 (36.2%)	48 (21.4%)	52 (23.2%)	15 (6.7%)
I would like more opportunities for individual supervision to be able to talk about the death of cancer patients.	2.75 (1.33)	47 (21.0%)	64 (28.6%)	40 (17.9%)	45 (20.1%)	28 (12.5%)
In general, I do not see any unmet needs with regard to dealing with the death of cancer patients.	2.37 (1.07)	52 (23.3%)	79 (35.4%)	56 (25.1%)	30 (13.5%)	6 (2.7%)

*Note: n* = 224.

Overall, 58.7% of participants disagreed with the statement, “In general, I do not see any unmet needs with regard to dealing with the death of cancer patients,” suggesting that the majority perceived the opposite to be true and recognized at least some unmet needs. More strikingly, 89.3% of participants endorsed at least one specific unmet need (e.g., wanting dedicated opportunities to discuss experiences with patient deaths), and 33.5% explicitly reported having personal unmet support needs. Regarding professional training, 37.9% of participants expressed a desire to be better equipped for handling patient deaths.

### 4.6 Exploratory Subgroup Analysis

Regarding number of colleagues, only minor differences in grief and overall distress were observed (Supporting Information S6: Table 5, Figures 1 and 2). A slight U‐shaped trend emerged for grief‐related distress (TRIG scores), with psycho‐oncologists reporting either no colleagues or more than ten colleagues showing numerically higher scores, though Kruskal‐Wallis tests revealed no significant differences.

For overall distress, participants with > 10 colleagues reported the highest levels (*M* = 6.14, MD = 6.5), while those with 1–5 colleagues reported the lowest (*M* = 4.75, MD = 5.0). Kruskal‐Wallis test indicated this difference was significant (*p* = 0.004), while all other pairwise comparisons did not reach significance.

For professional experience (Supporting Information S6: Table 5, Figures 3 and 4), grief and overall distress levels varied but showed no linear pattern. Highest levels of both outcomes were observed among those with < 1 year experience in psycho‐oncology. Psycho‐oncologists with 11–15 years of experience reported the lowest grief‐related distress but relatively high overall distress. However, Kruskal‐Wallis tests revealed no significant differences between experience groups for either outcome.

## 5 Discussion

The present findings suggest that psycho‐oncologists experience professional grief as a natural and generally adaptive emotional response to patient deaths. The emotions reported—such as sorrow, sadness, grief, and occasionally helplessness—are similar to personal grief, though the low to moderate levels of grief assessed with the TRIG‐D indicate that the intensity is lower [23]. Descriptively, the level of grief reported in our sample was somewhat lower than that observed in the Israeli sample investigated by Engler‐Gross et al. [2], potentially suggesting differences in experience or contextual factors. Several factors may contribute to this difference, including potential differences in healthcare system structures, measurement variations (the German TRIG‐D contains additional items) or sample composition (over 55% of the Israeli sample were social workers while our sample consisted predominantly of psychologists). However, these interpretations require comparative research to substantiate.

Previous findings on the impact of professional grief in healthcare professionals have been mixed [4, 5, 6, 29], with some studies highlighting predominantly negative effects and others noting potential for growth. In our sample professional grief was not accompanied by long‐term or global negative effects on personal or professional life. In fact, many participants reported positive psychological outcomes. Nevertheless, about one in four reported negative consequences in one assessed domain, indicating that professional grief may also entail burdens for some individuals.

Guilt, commonly reported among physicians [5], played a negligible role among psycho‐oncologists. This divergence may be attributable to the distinct nature of their professional responsibilities. Unlike physicians, psycho‐oncologists do not make medical decisions or bear the burden of treatment side effects or outcomes, which may shield them from feelings of moral responsibility or failure. These differences suggest that while professional grief is a common phenomenon across healthcare professions, its emotional expression and psychological consequences may vary depending on role‐specific factors, warranting further comparative research.

Coping mechanisms reported by participants were diverse, yet consistently rated as important. The presence of colleagues—both in informal and formal settings—emerged as a central support structure. Colleagues were not only identified as the most important source of support, but could also facilitate emotional relief, rated as one of the most important coping strategy. Compared to other healthcare professions, psychologists may be generally accustomed to emotional self‐reflection and open expression of one's own emotional responses, for instance through established formats such as clinical supervision [32, 33, 34, 35]. It is possible that this professional culture and training foster a greater readiness to acknowledge and process difficult emotions following patient deaths. This, in turn, might serve as a protective factor—potentially contributing to the comparatively lower levels of distress observed in this sample relative to findings from studies involving physicians or nurses [4, 7]. While this interpretation is plausible, it requires further investigation to be substantiated. Nevertheless, these findings underscore the value of peer‐based coping and highlight the potential for institutions to promote collegial exchange.

While colleagues were rated as the most important support resource, our subgroup analyses revealed a complex pattern: participants with 1–5 colleagues reported significantly lower overall distress than those with more than ten colleagues, with no differences in grief‐related distress. Research indicates that larger team size does not uniformly benefit performance and support [36, 37] and that team familiarity is particularly crucial during emotionally demanding situations [38]. Our findings suggest that close, familiar peer connections—whether in smaller teams or otherwise fostered—may be more protective than team size alone for managing professional grief.

This importance of collegial support may be particularly understood through the lens of disenfranchised grief [39]—grief that is not openly acknowledged, socially validated, or publicly mourned. Professional grief often remains invisible within healthcare settings, where emotional expression may be perceived as unprofessional [5]. Colleagues could provide a sanctioned space where this otherwise disenfranchised grief can be acknowledged and validated. This framework helps explain why peer support emerged as the most valued coping resource: it legitimizes an emotional response that broader professional and societal norms may dismiss.

Alongside social coping, individual strategies such as acceptance of death and commemoration rituals were perceived as highly beneficial and may promote self‐regulated adaptation to loss.

The frequent reports of positive impacts align with the concept of post‐traumatic growth—defined as positive psychological change following the struggle with a traumatic event [40]. Over three‐quarters of participants reported positive impacts on their sense of purpose and personal representation of life, and roughly one‐third reported positive impacts of professional grieving experiences on their personal and patient relationships. These substantial proportions suggest that professional grief may serve as a catalyst for growth rather than primarily a source of distress. This interpretation aligns with research linking perceived social support, feeling connected to others and reflective training to post‐traumatic growth [41, 42, 43, 44]. However, our cross‐sectional design limits causal inference. Future longitudinal studies should examine whether growth arises from coping resources, personal traits, or contextual factors. Viewing patient deaths as unavoidable distressing events, that yet yield an opportunity for growth may offer a useful framework: while grief responses are typically adaptive, insufficient coping resources or particularly distressing circumstances may still give rise to negative effects. As with post‐traumatic stress and growth, these reactions may represent two sides of the same coin.

### 5.1 Clinical Implications

From a clinical standpoint, fostering collegial support structures should be a key priority within psycho‐oncological and wider healthcare settings. Both formal (e.g., supervision) and informal (e.g., peer conversations) avenues should be actively promoted to support grief processing. Given that individual practices such as reflection and rituals also play a role, healthcare institutions may benefit from integrating grief‐sensitive approaches into their workplace culture (e.g., designated spaces for remembrance). Additionally, our findings suggest that psycho‐oncologists value being informed about patient deaths promptly and personally. Healthcare institutions should consider establishing clear communication protocols that enable timely notification, allowing professionals to process loss adequately. Lastly, the expressed need for better training in how to deal with patient deaths indicates that professional grief should be explicitly addressed within psycho‐oncology, or more general: psychology and psychotherapy curricula, and continuing education.

### 5.2 Research Implications

Further studies should examine potential risk factors for maladaptive or unprocessed professional grief. Identifying subgroups of professionals with higher vulnerability could help inform targeted support measures. Moreover, understanding protective factors present among psycho‐oncologists could guide interventions aimed at supporting other healthcare providers exposed to repeated patient loss. Our exploratory subgroup analyses suggest that grief and distress levels may vary especially in relation to number of colleagues, though no consistent pattern emerged. This warrants further investigation. Developing validated tools and norm values for assessing professional grief would also be essential to advance this field methodologically.

### 5.3 Strenghts and Limitations

This study is the first to explore professional grief among psycho‐oncologists in Germany using quantitative methods and one of the few in Europe. Strengths include a diverse national sample with varied professional experience, as well as the combined use of validated instruments and context‐sensitive, self‐developed items. However, several limitations must be noted.

First, the convenience sample may be subject to self‐selection bias and may not represent all psycho‐oncologists—particularly those less connected to professional networks or working in different institutional contexts. The study invitation's true reach remains unclear, as mailing list sizes were unknown and forwarding was encouraged but not trackable. Self‐selection may also occur earlier, as professionals entering psycho‐oncology might differ from others in their attitudes toward death and dying. Moreover, individuals experiencing severe professional grief might have refrained from participation due to stigma or fears of appearing “unprofessional,” despite guaranteed anonymity [2].

Second, the absence of validated cut‐off scores for the grief measures limits the interpretability of the reported low‐to‐moderate scores. The PBS‐D showed acceptable internal consistency, yet, as psychometric validation is ongoing, findings should be interpreted with caution. Comprehensive psychometric validation remains a task for future research.

Third, we did not assess how frequently individual patients were seen. As nearly 68% of respondents rated the length of the therapeutic relationship as relevant, this unmeasured variable could influence grief severity. Additionally, while we conducted plausibility checks on extreme self‐reported values (e.g., very high patient caseloads or death frequencies), such outliers may reflect either genuine diversity across varied psycho‐oncological settings or occasional reporting inaccuracies. However, our analyses confirmed these values did not substantively influence primary findings.

The cross‐sectional design precludes causal or temporal conclusions. Longitudinal research is needed to identify factors promoting adaptive versus maladaptive grief trajectories.

The predominance of female participants (91%) limits generalizability to male psycho‐oncologists, though this likely mirrors the broader psychology workforce.

Finally, generalizability beyond Germany may be limited due to structural differences in psycho‐oncological care underscoring the need for international replication and comparison.

## 6 Conclusion

This study offers an in‐depth empirical insight into the phenomenon of professional grief among psycho‐oncologists in Germany. The findings suggest that, for most, professional grief is an emotionally manageable and even constructive experience. While distressing in the short term, patient deaths may contribute to personal and professional growth. It remains unclear whether the experience of growth following patient deaths is primarily enabled by sufficient coping resources or influenced by other individual or contextual factors; further research is needed to clarify these pathways. At the same time, a substantial proportion of participants reported unmet needs, particularly related to support and training. These results highlight the importance of institutional structures that validate grief, facilitate peer exchange, and provide education on coping with loss. Further research is needed to better understand the variability in grief responses across healthcare roles and to develop evidence‐based strategies for supporting healthcare professionals in emotionally demanding fields.

## Author Contributions

I.S. is the responsible principle investigator of the study. I.S. and S.W. were involved in planning and preparation of the study. S.W. developed the questionnaire, while all authors critically discussed and revised the questionnaire. S.W. recruited participants and collected and analyzed the data. All authors interpreted the results. S.W. wrote the first draft of the manuscript. K.L., M.H., K.O., C.B., M.R., and I.S. critically revised the manuscript for important intellectual content. All authors gave final approval of the version to be published and agreed to be accountable for the work.

## Funding

The authors have nothing to report.

## Ethics Statement

This study was carried out according to the latest version of the Helsinki Declaration of the World Medical Association and respecting principles of good scientific practice. The local Ethics Committee of the University Medical Center Hamburg‐Eppendorf gave approval prior to investigation (approval number: LPEK‐0614). Study participation was voluntary and no foreseeable risks for participants resulted from the participation in this study. Participants were fully informed about the aims of the study, data collection and the use of collected data and written informed consent was obtained.

## Conflicts of Interest

The authors declare no conflicts of interest.

## Supporting information


Supporting Information S1

Supporting Information S2

Supporting Information S3

Supporting Information S4

Supporting Information S5

Supporting Information S6

## Data Availability

Data available on request from the authors.
