# Supplementary material for: Professional Grief Among Psycho‐Oncologists in Germany: A Cross‐Sectional Survey Study
Source: Psychooncology. 2025 Dec 5;34(12):e70355. doi: 10.1002/pon.70355 (PMC12680905; doi:10.1002/pon.70355)
Supplement: Supplementary file 3 — Supporting Information S3 [file PON-34-e70355-s005.docx]

**Supplementary file 3, Professional associations within the recruitment process**

**List of professional associations, who forwarded the study invitation to their members:**

- Psycho-Oncology Working Group of the German Cancer Aid’s Network of Comprehensive Cancer Centers (*Arbeitsgruppe Psychoonkologie des Netzwerks Onkologische Spitzenzentren – CCC*)
- State Consortium for Cancer Counselling Services (*Bundesarbeitsgemeinschaft für Krebsberatungsstellen e.V*.)
- Association for Continued Education in Psycho-Social Oncology (*Weiterbildung Psychosoziale Onkologie e.V.*)
- Psychosocial Working Group within the Society for Pediatric Oncology and Hematology (*Psychosoziale Arbeitsgemeinschaft in der Pädiatrischen Onkologie und Hämatologie - AG in der Gesellschaft für Pädiatrische Onkologie und Hämatologie e.V.*)
- German Cancer Association’s Consortium for Psycho-Oncology (*Arbeitsgemeinschaft Psychosoziale Onkologie der Deutschen Krebsgesellschaft*).
- Addition: members of a regional network of psycho-oncologists in the Hamburg metropolitan area (*Psychoonkologie Treffen – POT*)
