# Supplementary material for: Professional Grief Among Psycho‐Oncologists in Germany: A Cross‐Sectional Survey Study
Source: Psychooncology. 2025 Dec 5;34(12):e70355. doi: 10.1002/pon.70355 (PMC12680905; doi:10.1002/pon.70355)
Supplement: Supplementary file 5 — Supporting Information S5 [file PON-34-e70355-s003.docx]

**Supplementary file 5, Exclusion and imputation protocol^1,2^**

***Texas Revised Inventory of Grief – Present feelings subscale (TRIG)***

**Three participants (n= 3) had missing values on the TRIG scale, with all 16 items unanswered. Consequently, these individuals were excluded from the corresponding analyses, resulting in a final sample of n= 255 for this scale.**

***Professional Bereavement Scale***

***Short-term bereavement reactions (PBS-SBR)***

**For nine participants 17 items were missing on the respective scale, exceeding the 30% threshold for missing data. As a result, these cases were excluded from the scale-based analyses due to insufficient information to calculate a person-specific mean score. For one participant, one item was missing (respectively no more than 30% missing data). Therefore, a person-specific mean was imputed for the missing values, in line with the predefined criteria, resulting in a final sample of n= 249 for this scale.**

***Accumulated global changes (AGC)***

**For 13 participants the number of missing responses on the respective scale exceeded the threshold of 30% (i.e., more than 5 items missing). Therefore, these cases were excluded from the scale-based analyses.**

**For one participant one item was missing and for another participant three items were (for both: respectively no more than 30% missing data). Accordingly, person-specific mean scores were imputed for the missing items, following the established procedure, resulting in a final sample of n= 245 for this scale.**

^1^Christalle, E. *et al.* Through the patients’ eyes: psychometric evaluation of the 64-item version of the Experienced Patient-Centeredness Questionnaire (EPAT-64). *BMJ Qual. Saf.* https://doi.org/10.1136/bmjqs-2024-017434 (2024) doi:10.1136/bmjqs-2024-017434.

^2^Heymans, M. W. & Twisk, J. W. R. Handling missing data in clinical research. *J. Clin. Epidemiol.* **151**, 185–188 (2022).
